# A miRNAs catalogue from third-stage larvae and extracellular vesicles of *Anisakis pegreffii* provides new clues for host-parasite interplay

**DOI:** 10.1038/s41598-022-13594-3

**Published:** 2022-06-11

**Authors:** S. Cavallero, I. Bellini, A. Pizzarelli, B. Arcà, S. D’Amelio

**Affiliations:** Department of Public Health and Infectious Diseases, Sapienza University of Rome, Piazzale Aldo Moro 5, 00185 Rome, Italy

**Keywords:** Parasitology, miRNAs

## Abstract

Anisakids are widespread marine parasites of medical, veterinary and economic relevance. They infect marine natural hosts but humans can accidentally acquire the fish-borne zoonosis anisakiasis by ingesting infected raw fishes or mollusks. Among the several species described, *Anisakis pegreffii* is one of the main etiological agent of the disease, in particular in the Mediterranean area. Despite the growing evidence of miRNAs involvement in host-parasite interplay, and the emerging role of exosomal microvesicles in shuttling them between different cell types (and sometime across species), no information on miRNAs from any *Anisakis* species is presently available. In this study we isolated extracellular vesicles (EVs) released by *Anisakis pegreffii* infective third-stage larvae (L3) and analyzed by RNA-seq small RNAs from both L3 and EVs. We showed by nanoparticle tracking analysis that L3 release in culture medium particles of size compatible with the one of extracellular vesicles. A catalogue of 156 miRNAs from *A. pegreffii* was compiled by sequence comparison to evolutionary close species and miRNA prediction software. Using differential expression analysis, we identified a small number of highly abundant miRNAs in larvae and extracellular vesicles fractions whose potential biological relevance may deserve future investigation. Finally, *A. pegreffii* miRNAs were compared to those described in other parasitic helminths and predicted targets among human genes were searched, suggesting their potential involvement during infection.

## Introduction

Nematodes of the genus *Anisakis* are causative agents of anisakiasis, a fish-borne zoonotic disease characterized by mild to severe clinical signs related to gastric, intestinal, ectopic migrations and systemic allergic reactions. Humans acquire the infection through the ingestion of raw or undercooked fishes or molluscs harbouring the infective third-stage larval forms (L3). Due to several, not mutually exclusive, potential factors (as the natural large occurrence of L3 in teleosts, the unspecific symptoms which may lead to misdiagnosis and the lack of reliable and universal serological screening tests) the incidence of the disease in humans is likely to be largely underestimated^1^. Several studies in the last 30 years investigated the molecular systematics and population genetics of this parasitic family, revealing fundamental aspects related to speciation, ecology and biodiversity^2^. However, the current understanding of the pathogenic mechanisms occurring during the early and late phases of infections and the related clinical manifestations in humans is still very poor. The use of different cellular *in-vitro* models as fibroblasts^3^, human dendritic cells^4^ or human epithelial colonic cancer cells^5^ highlighted a range of modulatory activities triggered by *Anisakis*-derived products on host immunity and inflammations, as the upregulation of oxidative-stress, inhibition of apoptosis or inflammatory induction.

Recently, reports of *Anisakis* L3 co-occurrence with gastro-intestinal tumours and larval infection mimicking malignancy are increasing^6–9^. Considering the suggested potential association of exposure to *Anisakis* with an increased risk to develop gastric or intestinal adenocarcinoma^10^, further specific and detailed studies are needed to clarify its tumorigenic potential and more broadly to understand pathogenesis of anisakiasis.

Among molecules involved in host-parasite interplay ensuring parasite survival in inhospitable environments, excreted/secreted proteins represent the most studied class so far^11^. Extracellular vesicles (EVs) have been recently described as a shared inter-kingdom cross-talk system^12^. EVs are heterogeneous nanosized particles, including exosomes (30–130 nm in diameter), microvesicles and apoptotic bodies^13^. Exosomes are the most investigated category in many organisms, as they are released by donor cells to recipient cells being vehicles of bioactive messengers transported in a protected state. Extracellular vesicles have been recently described also in parasitic helminths^14^ and can be used by pathogens to deliver immunomodulatory or pathogenic molecules. In fact, exosomes transport a cargo of small non-coding RNAs (sncRNAs), lipids, DNA and proteins. Among ncRNAs, microRNAs (miRNAs) are the most extensively studied in the EVs cargo categories^15^ as they display selective profiles that may differ from the global miRNA contents of the parent cell or tissue, suggesting a modulatory role on target cells^16^.

MiRNAs are 21–23 nt in length, ubiquitously present in eukaryotes, showing also a tissue-specific expression patterns and playing relevant roles in post-transcriptional gene regulation by promoting degradation and/or translation repression of target messenger RNAs. Their role is crucial in every aspect of cell life, from cell growth and differentiation to apoptosis, development and immunity^17^. Firstly described in the free-living nematode *Caenorhabditis elegans*^18^, miRNAs are known for acting on endogenous genes, and pathogens may exploit miRNAs to target host genes. This is well known for viruses and bacteria^19^, and it recently emerged for parasites as well^20^. Such strategy may provide an evolutionary gain, as miRNAs, differently from proteins, do not evoke host immune responses.

In this context, the research on parasitic nematodes of medical and veterinary relevance is still on its infancy and, so far, limited information are available on miRNAs and extracellular vesicles from the family Anisakidae. Among similarities and differences across major groups of helminths for which data are available, common miRNA families were observed in nematodes EVs (mir-10 and let-7) and trematodes EVs (mir-71, mir-10, mir-190, let-7 and mir-2)^21^. Functional studies about EVs and/or miRNAs effects in-vivo and in-vitro are available only for four genera of parasitic nematodes, namely *Brugia, Heligmosomoides, Trichinella* and *Trichuris*^14^. To the best of our knowledge there is only one study available on *Anisakis* derived EVs, and it is essentially focused on the development of fluorescent staining for tracking in vivo uptake of EVs by recipient cells/organs^22^.

With the aim to get insights into the *Anisakis* miRNA repertoire, and to verify whether L3 release extracellular vesicles carrying miRNAs possibly involved in host manipulation, we performed a small RNA-Seq analysis on *Anisakis pegreffii* infective-third stage larvae and on its released extracellular vesicles and provide here the first catalogue of *Anisakis* miRNAs.

## Results

### Characterization of extracellular vesicles released from *Anisakis pegreffii* by nanoparticle tracking analysis

**Figure 1:**
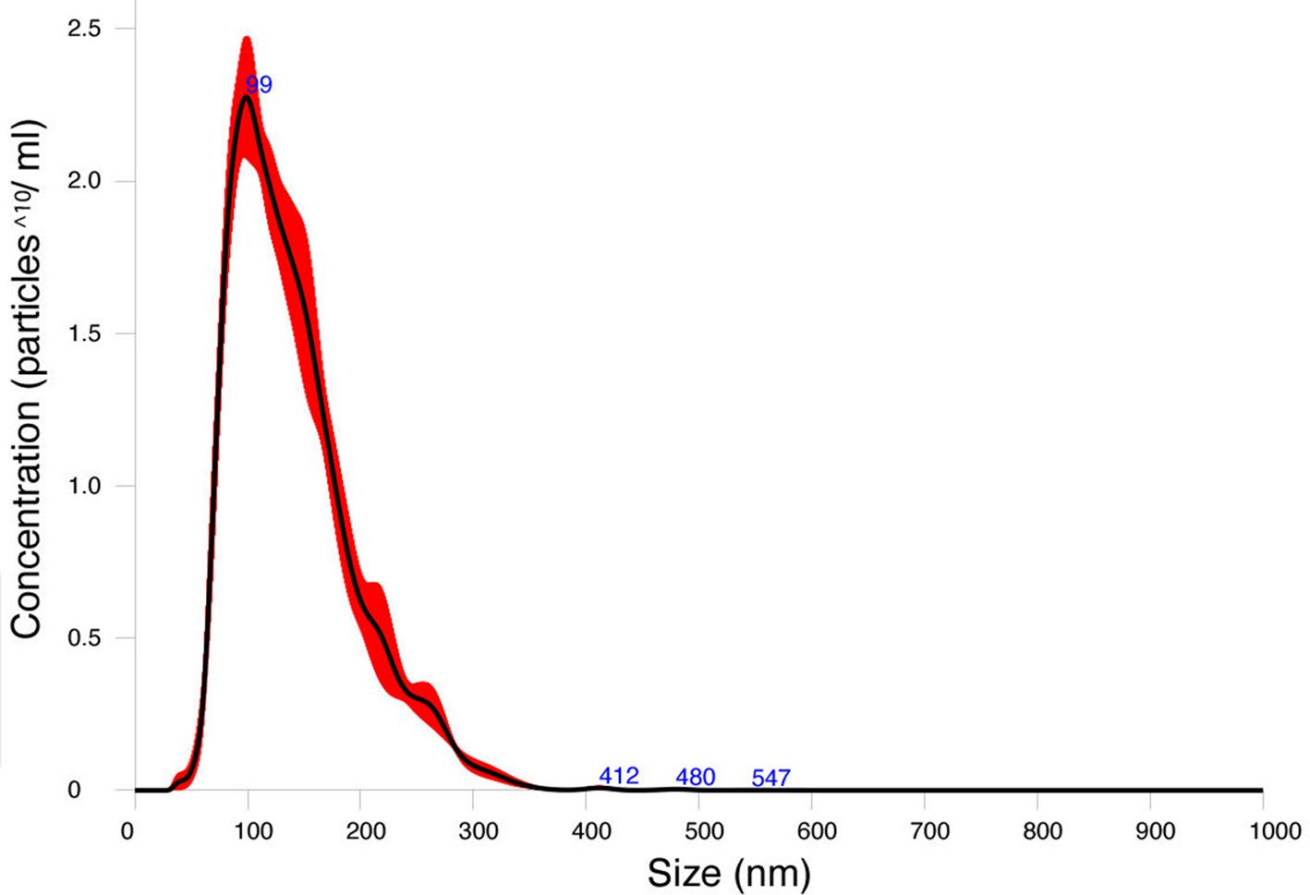
Finite track length adjustment (FTLA) Concentration/Size image for Nanoparticle Tracking Analysis (NTA) of extracellular vesicles secreted by third stage larvae of *A. pegreffii*.

As a first step, to verify whether *A. pegreffii* third-stage larvae (L3) release extracellular vesicles (= EVs hereafter), we incubated L3 in cell culture medium and, as described in the method section, treated their supernatants with an exosome precipitation solution. Size distribution and concentration of vesicles in this precipitated fraction were estimated by Nanoparticle Tracking Analysis (NTA) using the Nanosight technology. The analysis revealed the presence of particles that in large majority have a size (mean 140.5 ± 0.08 nm, mode 107.4 ± 4.6 nm; Fig. 1) fully compatible with extracellular vesicles, which usually have a diameter between 30 and 150 nm. Moreover, the mean number of particles observed per ml in the sample was 2.49 × 10^10^(± 1.16 × 10^9^). Details of NTA settings and results are available in the Supplementary Fig. 1.

### Deep sequencing of small RNAs from *Anisakis pegreffii* L3 and EVs

**Figure 2:**
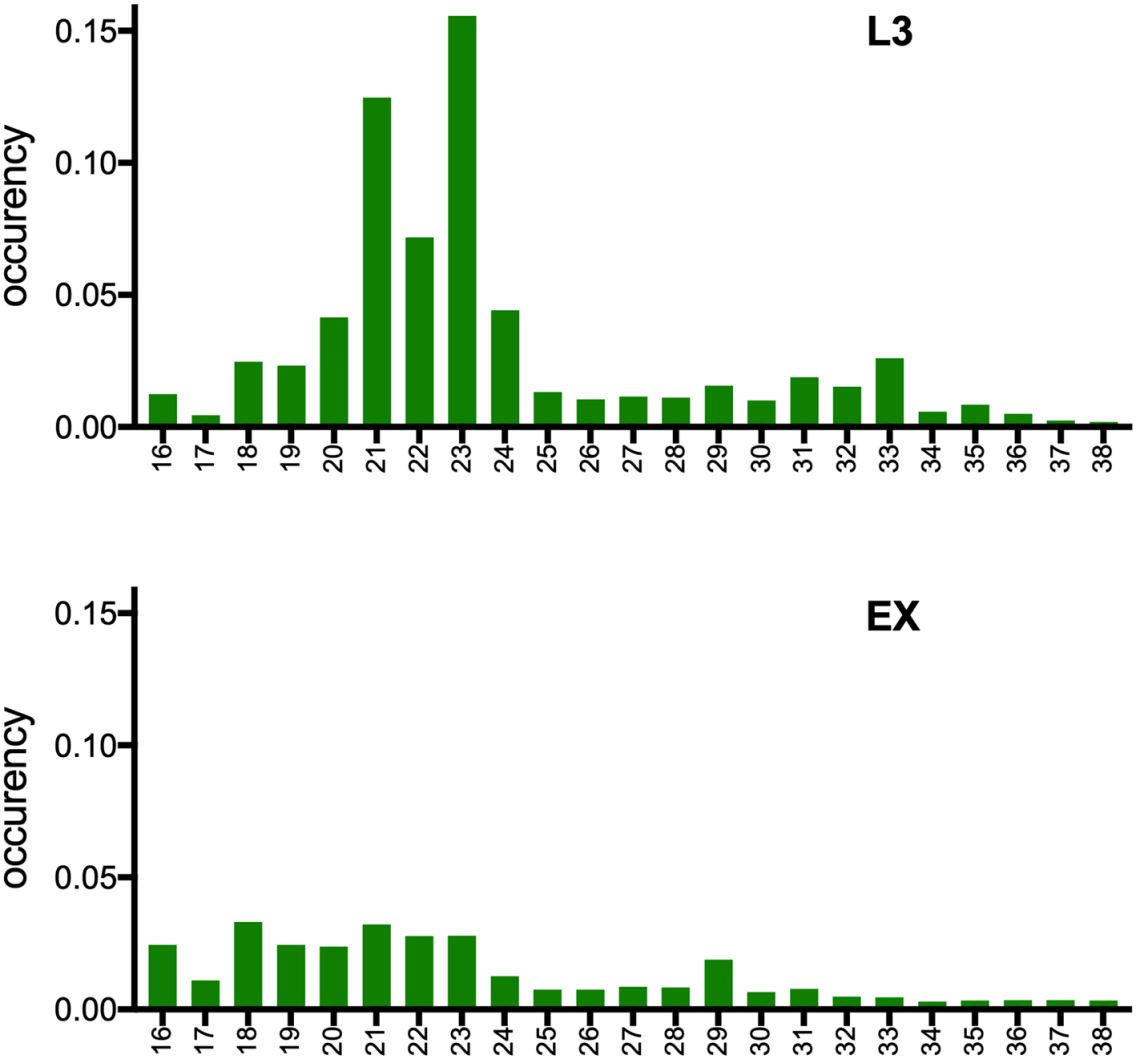
Bar plots with the frequency and size distribution of reads 16–38 nt in length mapping to the *A. simplex* genome (AS14) and subtracted of those mapping to rRNAs.

Small RNA fractions (< 200 nt) were extracted from *A. pegreffii* L3 and EVs (three biological replicates per sample type) and used for the construction of 6 small RNA libraries suitable for Illumina high-throughput sequencing. A total of ~ 170 million raw reads (L3: ~ 100 million; EVs: ~ 70 million reads) were obtained as indicated in Table 1. After quality filtering, adapter trimming and size selection (≥ 14 nt), approximately 140 million reads were retained and mapped to the *A. simplex* genome (AS14). Reads mapping to AS14 (a total of ~ 55 corresponding to the 38.74% of the reads), excluding those representing ribosomal RNAs, were analysed for their size distribution (Fig. 2). In third stage larvae most reads were in the range of 20–24 nt in length, with two peaks at 21 nt and 23 nt. Although these lengths are fully compatible with the mean size of miRNAs, this bimodal distribution is not typical in small RNA-seq studies. For this reason we analyzed the ten most abundant sequences in the 21 nt and 23 nt peaks and found that they all represented miRNAs and accounted for 84% and 78% of the peak content, respectively. Among highly represented miRNAs were miR-100, miR-71, miR-9, miR-5358a and miR-5360. Extracellular vesicles enriched fractions showed a substantially different size distribution with no evident peaks and a higher frequency of reads 18–23 nt in length. Analysis of the most abundant sequences in this range also showed that miRNAs were highly represented, confirming the overall suitability of our RNA-seq libraries for the planned miRNA analysis.

**Table1:** RNA-seq results from the sequencing of the small-RNA fraction isolated from infective third-stage larvae of *Anisakis pegreffii* and its released extracellular vesicles. Data on raw reads, reads passed cutadapt, reads mapping to the *Anisakis simplex* genome and to rRNAs are reported. Numbers indicate million reads.

Sample	Raw reads	Filtered reads (passing cutapadpt)	AS14 genome mapped	rRNAs
L1	30.158	23.879 (79.2%)	10.929 (45.77%)	8.043 (73% of genome mapped)
L2	33.620	27.311 (81.2%)	13.966 (51.14%)	10.845 (77% of genome mapped)
L3	37.771	29.741 (78.7%)	13.804 (46.41%)	10.675 (77% of genome mapped)
Ex1	27.284	24.641 (90.3%)	4.890 (19.85%)	4.111 (84% of genome mapped)
Ex2	19.089	15.933 (83.5%)	3.146 (19.74%)	2.425 (77% of genome mapped)
Ex3	23.817	20.666 (86.8%)	8.345 (40.38%)	7.456 (89% of genome mapped)
Total	171.739	142.171	55.080 (38.74%)	43.555 (79% of genome mapped)

### *A. pegreffii* miRNAs prediction and counting

Given the absence of information on miRNAs from any *Anisakis* species, we used two complementary approaches to obtain a list of putative miRNAs from *A. pegreffii*. First, we retrieved from miRBase^23^ all the available miRNAs from the pig roundworm *Ascaris suum*, which is the species with experimentally validated miRNAs evolutionary closer to *Anisakis* species. We then used these miRNAs to search for putative orthologues in the *Anisakis simplex* genome and, using this in silico approach, we obtained a list of 97 hypothetical *A. simplex* miRNA precursors and 115 mature miRNAs (dataset 1). In a second approach, as described in detail in the method section, reads from our samples were used to search the *A. simplex* genome using the miRNA prediction software miRDeep*. This way we obtained a list of 150 putative mature (and corresponding precursors) miRNAs from *A. simplex* (dataset 2). By merging these two datasets, we obtained a final non-redundant list of 206 putative *Anisakis* mature miRNAs. Alignments of reads from our L3 and EVs samples to this list, considering only miRNAs with reads in two replicates of at least one sample, provided evidence for the expression of 156 mature miRNAs. Among these, 67 (43%, from 46 miRNA precursors) were putative orthologues of *A. suum* and were renamed according to the current miRNAs nomenclature, as ape-miR- followed by the identification codes used for *A. suum*. The remaining putative novel 89 miRNAs (57%) were named as novelMiR followed by the identification number assigned by the miRDeep* software. Most of the miRNAs were observed in the L3 samples (126) while 30 were observed also in EVs samples and no miRNAs were reported exclusively in the EVs samples. Raw data from RNA-seq of *A. pegreffii* small-RNAs has been deposited to SRA under the Bioproject PRJNA786753. The list of 156 *A. pegreffii* miRNAs with related features is available as Supplementary Table 1.

**Figure 3:**
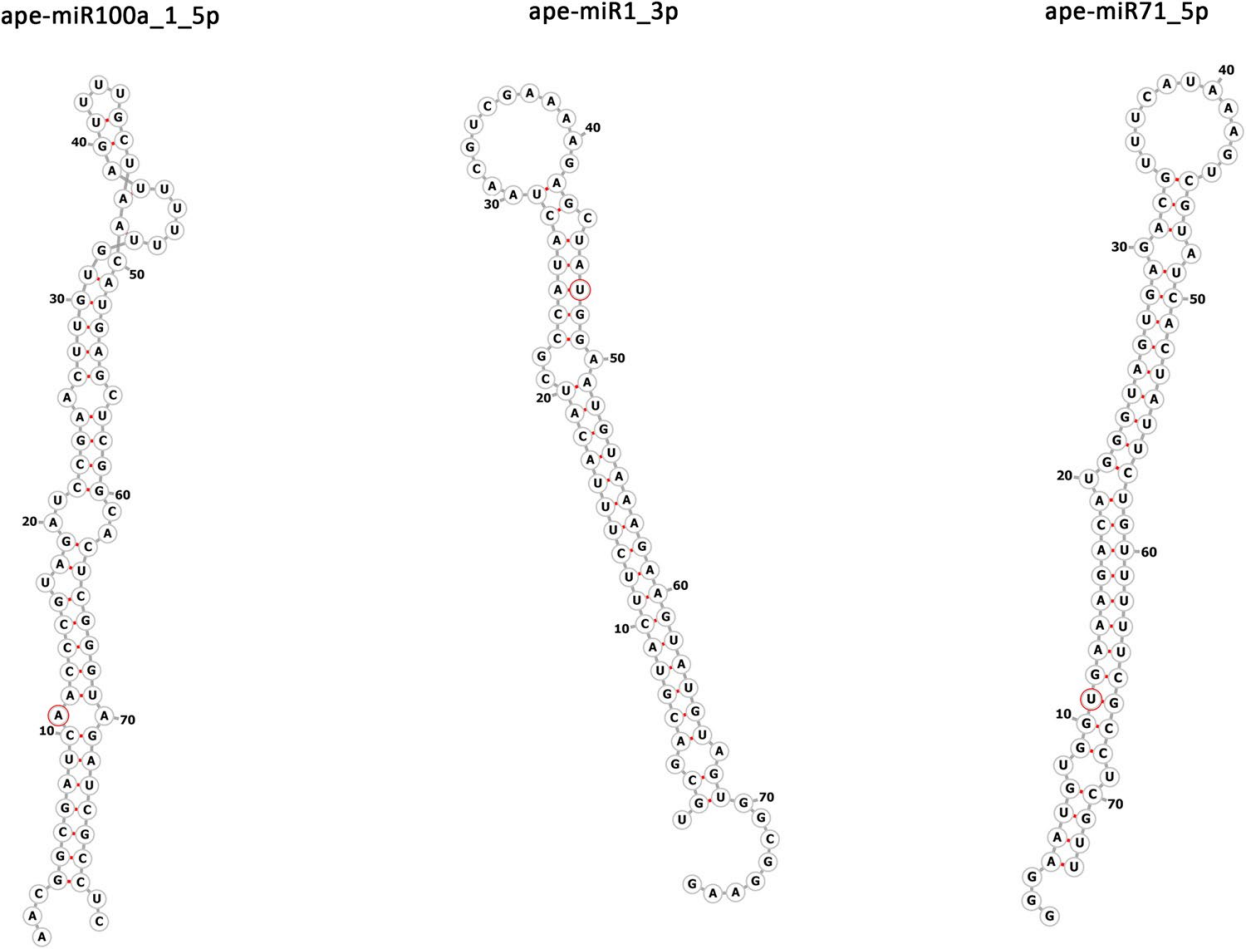
Secondary structures of the three most abundant miRNAs observed in the infective third stage larvae of *A. pegreffii* with the first nucleotide of matures sequence indicated in red, according to RNA fold.

Four miRNAs (ape-miR-100a-5p, ape-miR-1-3p, ape-miR-71-5p and ape-miR-9-5p) were by far the most abundant in L3, with > 100,000 mean CPM. The remaining miRNAs could be classified in four additional categories according to CPM values: (i) > 10,000 n = 8; (ii) > 1,000 n = 15; (iii) > 100 n = 33; (iv) ≥ 10 n = 63). Interestingly, ape-miR-100a-5p was also the most abundant miRNAs in EVs (629,841 mean CPM, 8720 mean counts). The 20 most abundant miRNAs identified in L3 are listed in Table 2. The predicted secondary structures for the first three most abundant miRNAs are shown in Fig. 3.

**Table 2:** Twenty most abundant miRNAs in infective third-stage larvae (L3). Sequence and mean count per million in L3 and EVs are reported.

ID	sequence	L3 CPM	EVs CPM
ape-miR-100a-5p	AACCCGTAGATCCGAACTTGTGTT	251,942.14	629,840.86
ape-miR-1-3p	TGGAATGTAAAGAAGTATGTA	250,143.69	59,391.08
ape-miR-71-5p	TGAAAGACATGGGTAGTGAGACG	136,994.96	152,149.96
ape-miR-9-5p	TCTTTGGTTATCTAGCTGTATGA	125,915.79	38,209.08
ape-miR-100b-5p	AACCCGTAGAATCGAATTTGTGTT	45,659.44	15,982.75
ape-lin-4-5p	TCCCTGAGACCTCTGCTGTGA	20,295.25	36,488.92
ape-miR-81a	TGAGATCATTGTGAAAGCTCTT	19,617.25	12,951.57
ape-miR-5361-5p	TGGGATATCTTGGAAGTTTTCA	19,199.65	16,499.62
ape-miR-57-5p	TACCCTGTAGTACCGAGCTGTGTTT	18,180.44	2,969.04
ape-miR-5358a-3p	TACCCGTAATTGCCATGACTGTT	13,532.42	6,932.50
ape-miR-50-3p	TGATATGTCTGGTATTCTTGGGTT	11,022.90	2,728.67
ape-miR-5364-3p	AGAGGTATTGTTTATTGGCTAA	10,641.77	4,231.83
ape-miR-34-5p	TGGCAGTGTGGTTAGCTGGTTGT	7,053.78	3,350.12
ape-miR-5360-5p	ACGAATCGTCGAATCGGATGTCT	5,998.75	154.95
ape-novel-miR-124	TACTGGCCTTCTAAACTCAACGA	5,616.68	6,138.17
ape-miR-228-5p	AATGGCACTAGATGAATTCACGG	4,682.19	77.90
ape-miR-9-3p	ATAAAGCTGGACGACCGAAGTAA	4,254.67	2,519.03
ape-miR-1822-3p	GAGCTGCCCTCTGAAAGATTGA	3,666.48	3,316.96
ape-novel-miR-185	GTAGCGACGTGGAGCACGA	2,770.05	261.70
ape-novel-miR-72	GTGGTTAGGATTTGCGGCTCTCA	2,445.47	2,429.88

**Figure 4:**
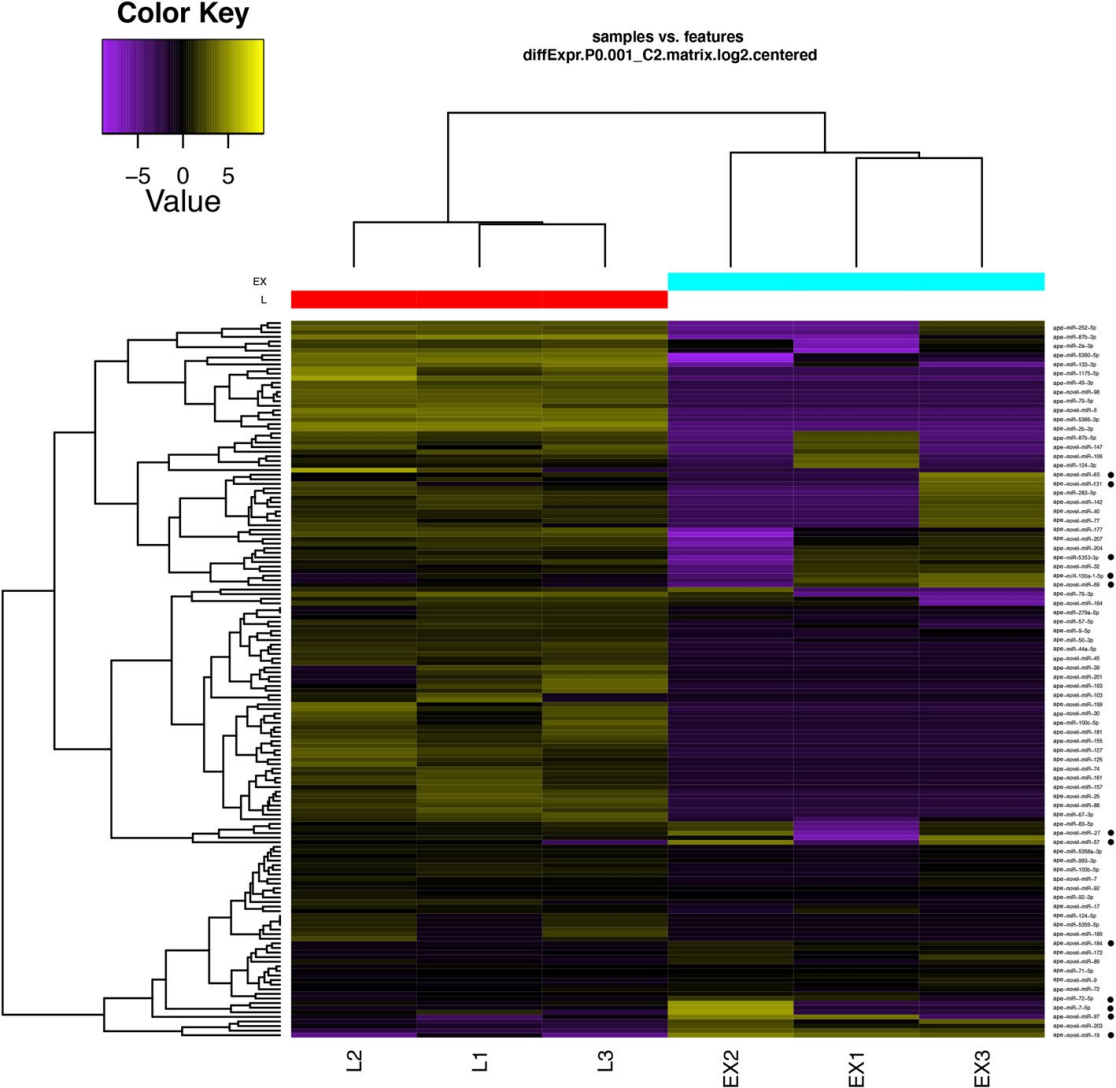
Heatmap and hierarchical clustering of mature miRNAs expression profiles of the third stage larvae of *A. pegreffii* (L) and of its released extracellular vesicles (EX). Each line corresponds to the mean-centered log2-transformed CPM, colored according to upregulation (yellow) and downregulation (violet). Upregulated miRNAs in EVs sample are indicated with a black dot.

The mature miRNAs correlation and cluster analyses confirmed the overall good quality of replicates, showing a higher homogeneity in the larval sample than in extracellular vesicles sample, which maintain their clustering trend despite the higher variation, probably due to sample normalization effect on triplicates (Supplementary Fig. 2). The expression heatmap from matures miRNAs highlighted groups with specific profile signatures, of which a few corresponded to enriched miRNAs in EVs samples (Fig. 4).

**Figure 5:**
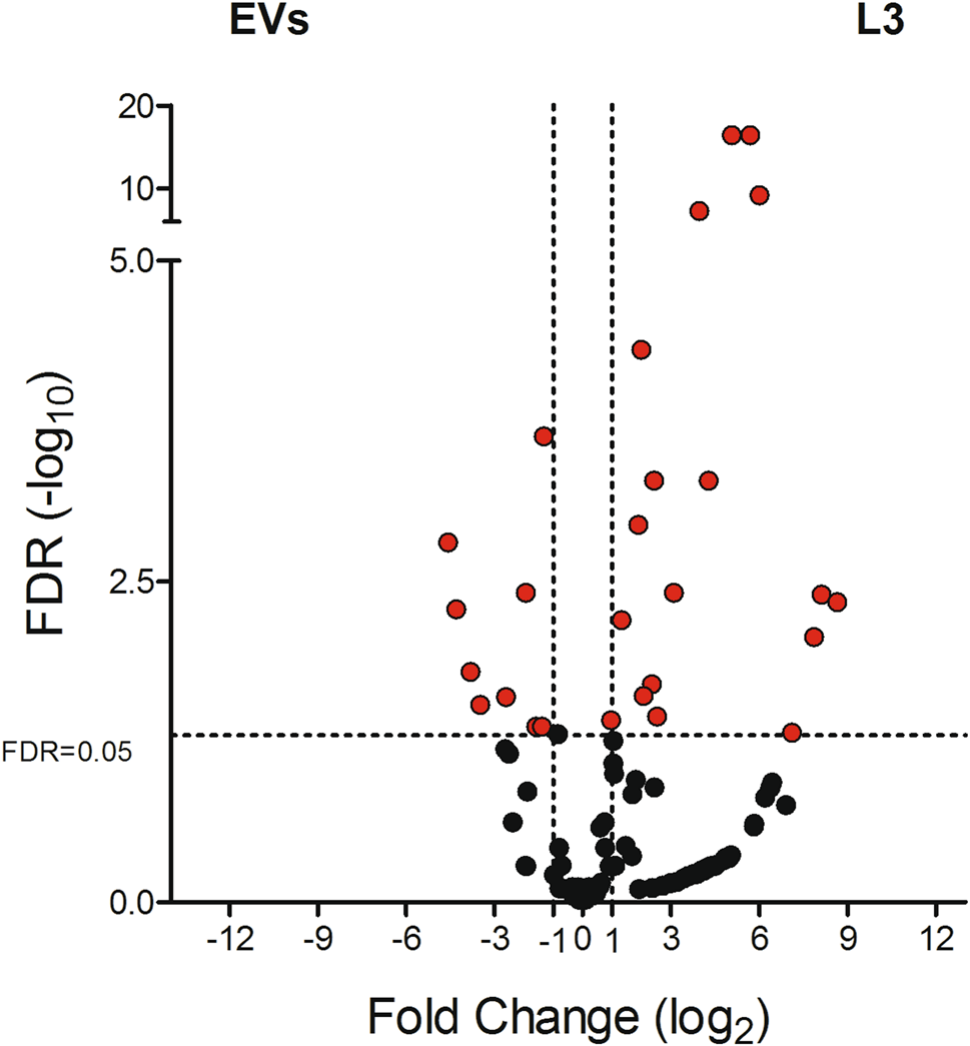
Volcano plot with the differential abundance of miRNAs in the pairwise comparisons between third-stage larvae (L3) and in the extracellular vesicles-enriched fraction (EVs) of in *Anisakis pegreffii*. The log2 fold change (FC) versus the negative log10 of false discovery rate (FDR) as calculated by the Fisher’s exact test are reported. Vertical dotted lines mark logFC = 2, horizontal dashed lines mark FDR threshold equal to 0.05.

Sample-specific miRNA enrichment was evaluated by pairwise comparisons between the two samples (L3 vs EVs): fold change (FC) and false discovery rates (FDR) were calculated to provide statistical validation (Supplementary Table 2). Using as threshold parameters |log2(FC)|> 1 and FDR < 0.05, we found that 38 miRNAs were differentially expressed, with 26 upregulated in L3 and 12 in EVs as shown in the Volcano plot (Fig. 5). Number of differentially expressed miRNAs applying progressively more stringent statistical thresholds are shown in Table 3.

**Table 3:** Number of differentially expressed miRNAs in *Anisakis pegreffii* larvae and released extracellular vesicles, according to three levels of statistical significance.

FDR	L3	EVs	TOT
< 0.05	26	12	38
< 0.01	23	7	30
< 0.001	14	4	18

### Stem and Loop RT-PCR validation of miRNAs

With the aim to confirm the presence of miRNAs in the samples studied, a list of ten miRNAs has been selected for their experimental validation in Stem and Loop RT-PCR. The list has been elaborated based on abundance category for L3 (three representative of the first categories of abundance, as > 100,000 mean CPM; > 10,000 mean CPM and > 1,000 mean CPM), on the novelty and on their differential expression (upregulated in EVs). All the ten selected miRNAs were successfully amplified and confirmed using Real Time Stem and Loop PCR (novel-miR-19, miR-7-5p, novel-miR-65, novel-miR-27, miR-72-5p, novel-miR-184, miR-100a-1-5p, lin-4-5p, miR-1-3p, novel-miR-131).

The following average of Ct were obtained for the larval sample: 17.5 for miR-100a-5p; 18 for miR-1-3p; 19.1 for novel-miR-131; 21.75 for miR-72-5p; 28.6 for novel-miR-19, 29.6 for miR-7-5p; 33.1 for novel-miR-184; 35.2 for lin-4-5p; 36.5 for novel-miR-65 and novel-miR-27.

The following average of Ct were obtained for the extracellular vesicles: 26 for novel-miR-131; 26.9 for novel-miR-19; 27.5 for miR-1-3p; 28.3 for miR-100a-1-5p; 29.6 for miR-72-5p; 31.8 for novel-miR-184; 34.4 for miR-7-5p; 35 for novel-miR-27 and lin-4-5p; 36.8 for novel-miR-65. A barplot of mean Ct values and SE obtained for L3 and EVs assays is available in Supplementary Fig. 3.

### Target analysis and seed conservation in parasitic helminths

The complementarity between the 3′-UTR of the mRNA target and the miRNA seed region (nucleotides 2–8) is a crucial feature of miRNA-mRNA interaction. Identity of the entire miRNA or of the seed region may be indicative of evolutionary conservation of its function.

Comparison of *A. pegreffii* miRNAs to those from other helminths as Nematoda (*A. suum, Trichuris muris, B. malayi, Nippostrongylus brasiliensis, Heligmosomoides polygyrus, Haemonchus contortus),* Trematoda (*Fasciola hepatica, Schistosoma mansoni, Schistosoma japonicum)* and Cestoda (*Dibothriocephalus dendriticus, Mesocestoides corti, Taenia crassiceps, Taenia asiatica, Echinococcus multilocularis*) showed different levels of conservation. A complete seed conservation was reported between miRNAs from *A. pegreffii* with those of parasitic nematodes *A. suum* as shown in Table 4 (85% of the most abundant in L3 and 46% of EVs enriched) followed by *B. malayi* (75% of the most abundant in L3 and 31% of EVs enriched). Additionally, complete seed conservation and miRNAs homology were observed with other helminths as trematodes and cestodes as well as with human miRNAs, suggesting a potential functional role conserved across phylogenetically distant taxa.

**Table 4:** List of ten selected *Anisakis pegreffii* abundant miRNAs in larvae and in extracellular vesicles, together with putative orthologues miRNAs from other parasitic helminths with conserved seed region, and human miRNAs putative orthologues. The last two columns include *Anisakis pegreffii* miRNAs predictive targets in human genome according to miRDB and their ID according to NCBI database. Asterisks indicate a miRNAs enriched in exosomes, according to literature. (Underlined miRNAs are abundant both in L3 and in EVs list).

*Anisakis pegreffii* miRNAs	miRNAs in other helminths	Human miRNAs	Target—putative role	GeneID NCBI
miR-100a-5p	asu-miR-100b-5p bma-miR-100a bma-miR-100b bma-miR-100c bma-miR-100d hpo-miR-100-5p*	hsa-miR-100-5p	**TRIB2**—interact and modulate the activity of signal transduction pathways in physiological and pathological processes. This Tribbles member induces apoptosis of cells mainly of the hematopoietic origin	28951
miR-1-3p	asu-miR-1-3p str-miR-1-3p hco-miR-1-3p hpo-miR-1-3p fhe-miR-1-3p	hsa-miR-1-3p	**MMD**—monocyte to macrophage differentiation associated protein	23531
miR-71-5p	asu-miR-71-5p bma-miR-71 str-miR-71-5p hpo-miR-71-5p hco-miR-71	No match	**NAA25**—auxiliary subunit of the heteromeric N-terminal acetyltransferase B complex	80018
miR-9-5p	asu-miR-9-5p bma-miR-9-5p str-miR-9-5p hco-miR-9 hpo-miR-9-5p	hsa-miR-9-5p	**PRTG**—protogenin, member of the immunoglobulin superfamily	283659
lin-4-5p	asu-lin-4-5p bma-lin-4 str-lin-4-5p hco-lin-4 hpo-lin-4-5p cel-lin-4-5p	hsa-miR-125b	**ARID3B**—family of DNA-binding proteins with roles in embryonic patterning and cell lineage, cell cycle control, transcriptional regulation and chromatin structure modification **STARD13**—regulation of cytoskeletal reorganization, cell proliferation, cell motility, and acts as a tumor suppressor in hepatoma cells **BMF**—apoptotic activator **FREM1**—co-receptor of the interleukin 1 receptor family contributing to the control of inflammatory response activation	10620 90627 90427 158326
miR-5358a-3p	asu-miR-5358a-3p	No match	**TRIB2**—interact and modulate the activity of signal transduction pathways in physiological and pathological processes. This Tribbles member induces apoptosis of hematopoietic cells	28951
miR-5364-3p	asu-miR-5364-3p bma-miR-5364	No match	**STARD13**—regulation of cytoskeletal reorganization, cell proliferation, cell motility, and acts as a tumor suppressor in hepatoma cells	90627
novel-miR-19	No match	hsa-mir-1322 hsa-mir-4502	**FAM83C—**involved in regulating MAPK signaling in cancer cells	128876
novel-miR-97	No match	No match	**LINGO1—**leucine rich repeat and Ig domain	84894
miR-5353-3p	asu-miR-5353-3p	No match	**NECAP2—**member of the adaptin-ear-binding coat-associated protein family. Studies of a similar protein in rat suggest a role in clathrin-mediated endocytosis	55707

The most abundant miRNA in both larvae and extracellular vesicles is ape-miR-100a-5p that shows complete homology to miR-100 from other parasitic helminths and humans. It belongs to the miR-10 family, which besides miR-100 also includes miR-51, miR-57 and miR-99 (Rfam RF00104). The predicted putative gene target of ape-miR-100a-5p is TRIB2, a crucial gene for regulation of apoptosis and thymocyte cellular proliferation^24^, and its dysregulation was found associated with tumors, including colorectal cancer^25^.

**Figure 6:**
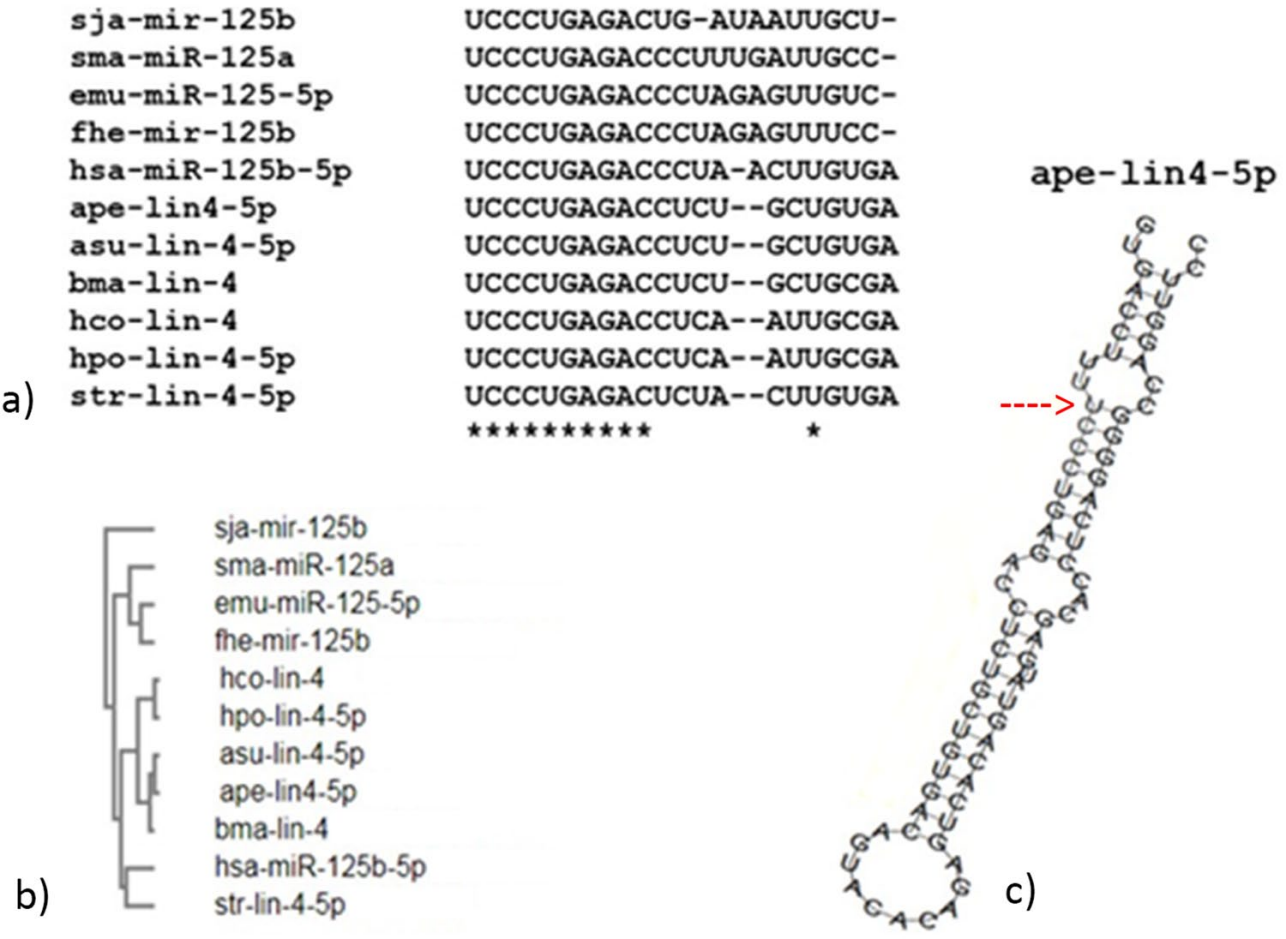
Alignment of miRNA-125 and lin-4 from several helminths and human is reported in (**a**) (sja: *S. japonicum*; sma: *S. mansoni*; emu: *E. multilocularis*; fhe: *F. hepatica*, hco: *H. contortus*; hpo: *H. polygyrus*; asu: *A. suum*; ape: *A. pegreffii* present study; bma: *B. malawi*; hsa: *H. sapiens*; str: *S. ratti*). Complete identity is highlighted by an asterisk, while dashes indicate nucleotide deletions. The dendrogram of aligned miRNAs is shown in (**b**) and hairpin structure of ape-lin4 with the first nucleotide of matures sequence indicated with a red arrow is available in (**c**), according to RNA fold.

Another abundant miRNA is lin-4-5p: originally identified in *C. elegans* in relation to developmental timing^26^, it shows complete seed identity with miR-125 of several parasitic helminths and humans. The dendrogram obtained from the comparison of the available orthologues showed similarities between ape-lin-4 in *A. suum* and *B. malayi* (Fig. 6). miR-125 seems to be involved in fundamental aspects of parasitic infection: *F. hepatica* miR-125b (fhe-miR-125b) was the most abundant miRNA trafficked by EVs observed in peritoneal macrophages during the early phase of infection. Given the homology with the mammalian miRNA hsa-miR-125b, it has been suggested the hijacking of the miRNA machinery as a parasitic strategy to control host innate cell function^27^. Ape-lin-4-5p showed several interesting potential gene targets: ARID3B involved in crucial cellular processes as transcriptional regulation^28^, STARD13 involved in cell proliferation and tumor suppression and in particular, miR-125b induces metastasis by targeting STARD13 in *in-vitro* breast cancer cells^29^. Additional gene targets are BMF, related to apoptosis activation^30^ and FREM1, a gene related to IL-1 inflammatory response^31^.

Among miRNAs selectively packaged into extracellular vesicles, novel-miR-19 showed no match with other helminths but a match with human miRNAs was observed (Table 4): its putative gene target is FAM83C, which is involved in regulation of MAPK signaling in cancer cells^32^.

## Discussion

Investigations based on high-throughput sequencing techniques as genomic, transcriptomic and proteomic approaches are providing important insights into nematodes parasitic biology as well as parasite-host interactions. In this scenario, small non-coding RNAs are a promising category of molecules with biological functions^16,33^. Among these, helminths secreted miRNAs could be fundamental in shaping host-parasite interactions and are potential targets for diagnosis and therapy^14^. Some of them are selectively packaged into extracellular vesicles. The interest in EVs is growing due to the increasing studies demonstrating their crucial involvement in intercellular communication, modulation of immune responses, pathology, and their fundamental regulatory role in host-parasite interface during helminthic infections^14^. Parasitic helminths are of particular interest, because they usually determine chronic but rarely lethal infections by manipulating host immune system, thus allowing the survival in an adverse niche. Despite their socioeconomic and medical impact, research on this field is still on its infancy, and most of the efforts to characterize nematode miRNAs focused on whole-worm extracts and not on miRNA populations secreted by parasites.

Here, we provide the first miRNAs catalogue from the zoonotic species *A. pegreffii* with 156 predicted matures, a widespread parasitic nematode of medical and economic concerns. Moreover, we confirm the ability of anisakids to releases EVs during its infective stage (L3). Size of *A. pegreffii* EVs are in agreement with the only available evidence about the genus *Anisakis* sp.^22^ and with other parasitic helminths as *A. suum, B. malayi, H. polygyrus, T. suis* and *F. hepatica*^34–37^.

So far, the two registries for miRNAs (miRBase and MirGeneDB) contains a scarce number of miRNAs from parasitic nematodes (e.g. *A. suum* 189 matures and 97 hairpins*, B. malayi* 166 matures and 157 hairpins*, S. ratti* 208 matures and 106 hairpins*, H. polygirus syn* 486 matures and 246 hairpins*, H. contortus* 194 matures and 188 hairpins). Comparisons with material retrievable from public repositories showed 43% (n = 68) of *A. pegreffii* miRNA catalogue with high level of homology with the phylogenetically related species as *A. suum* and in around 30% (n = 47) a partial conservation of sequence also with other helminths or organisms was observed. The remaining 25% of miRNAs (n = 41) were observed only in this parasitic organism based on current sequence data, and did not have homologues in any species, being putatively classified as “*bona fide*” species-specific for *A. pegreffii*. It is possible that additional non-conserved miRNAs could be present in *A. pegreffii* and these could be identified by analysing additional life stages and/or with the support of a reliable species-specific genome. In fact, the *A. pegreffii* reads from RNA-seq were, of necessity, mapped to the *A. simplex* genome, therefore additional *A. pegreffii*-specific sequences may have been undetected in the proposed miRNAs list. Similar issues were observed in previous studies we performed for the entire repertoire of transcripts of *A. simplex* sensu stricto and *A. pegreffii*, showing ranges of only 80–85% of genome mapping^38,39^, even for specimens belonging to the same species, thus suggesting the low annotation reliability of the available *A. simplex* genome.

Among the conserved miRNAs, one of the two common miRNAs of nematodes with crucial role in development (lin-4 and let-7) was not observed here: let-7 was not included in the final version of the catalogue according to its very low abundance and its amount of variation compared to known let-7 sequences. A similar evidence was reported in *H. contortus*^40^. The scientific community is still questioning whether miRNAs from body fluids play biological functions, as the concentration of extracellular miRNAs may be too low to exert in vivo effect^41^.

Half of miRNAs enriched in the extracellular vesicles fraction were novel. Since previous works used the number of sequence reads of a particular miRNA from deep-sequencing as an indication of molecular abundance^40,42^, some miRNAs found at high abundance in infective third-stage larvae were abundant also in the extracellular vesicles fraction (miR-100a-5p, miR-1-3p, miR-71-5p, si-miR-9-5p). In particular, ape-miR-100a-5p is the only miRNAs among the four highly abundant that is upregulated in EVs (FC = 2.49, FDR = 0.00023) and such large presence may indicate a biological role. Another abundant miRNA is ape-lin-4, found both in larvae and EVs. It has been suggested that a dysregulation of miR-100-5p may be related with several types of human cancers [43–45]. Similarly, the potential targets of ape-lin-4 are genes involved in cellular proliferation, tumor suppression and induction of apoptosis.

Other miRNAs found differentially expressed and enriched in EVs were mostly novel and not very abundant, but their validity confirmed by Stem and Loop RT-PCR and their selective package into EVs may suggest a potential role in host-parasite interplay. Previous studies identified miR-71 and miR-100c as common markers in the sera from hosts infected with *B. malayi, Dirofilaria immitis* and *Loa loa*^34^.

Studies on exosomal miRNAs from human parasitic nematodes have been conducted only on adults and L3 of *Brugia*^34,46^ and *Ascaris*^37^. Promising evidences were obtained with animal models and a role of exosomal miRNAs in targeting host genes associated with immunity and inflammation has been identified for the intestinal nematode of rodents *Heligmosomoides*^47^. In fact, administration of nematode exosomes to mice suppresses Type 2 innate responses and eosinophilia induced by the allergenic fungus *Alternaria,* by silencing Il33r and Dusp1. Similarly, Hansen and colleagues^37^ found IL33 gene as one of the putative targets of *A. suum* derived miRNAs. This gene was deregulated in jejunal mucosa of pigs infected with *A. suum*^48^.

Soluble products from *Trichuris suis* can modulate immune response via direct interactions but also indirectly by eliciting the release of EVs from bone marrow-derived macrophages that exert anti-inflammatory effects on recipient cells, by suppressing TNFα and IL-6 release and deregulating redox homeostasis^49^. Other helminths showed the ability to regulate host macrophages and modulate the host immune response to facilitate parasite survival using EVs and miRNAs: adults of *S. japonicum* secreted EV miR-125b and bantam, which increased macrophage proliferation and TNF-α production by regulating the corresponding targets including Pros1, Fam212b, and Clmp^50^.

Here, the experimental settings for EVs release by L3 were selected to mimic human body/host conditions (body temperature 37 °C). We would like to point out that humans are accidental hosts, and therefore interactions taking place between *Anisakis* and humans are not the result of natural evolutionary processes, such as co-evolution and/or co-adaptation. Nevertheless, extracellular vesicles miRNAs released by *Anisakis* L3 may be involved in pathogenic condition and clinical outcomes, and the analyses of putative miRNAs targets may shed light in their potential pathogenic effect.

Most of the identified miRNAs were common in the two classes of material analysed, namely the infective larval stage and its released extracellular vesicles, as expected. However, a group of 12 miRNAs was enriched in the latter and gene target analyses using human genome as query revealed several predicted gene targets of high interest. These are involved in crucial processes during infections, as cellular proliferation and/or differentiation during the shift from innate to adaptive immune response, apoptosis and inflammation, commonly associated with the functions of extracellular vesicles and exosomes^14^.

In conclusion, the present study provides the first evidence of EVs miRNAs content released by *A. pegreffii* infective larvae: altogether, the results suggest larval and EVs associated miRNAs may play an important role in the host–parasite interplay, given the wide conservation of sequences among parasitic helminths.

## Methods

### Fish and parasites collection

Third stage larvae (= L3) of parasitic nematodes belonging to *Anisakis* genus were collected from fishes purchased at market in 2019–2020. No live fishes have been involved in the study. In brief, visceral body cavity of 10 specimens of *Merluccius merluccius* and 50 specimens of *Engraulis encrasicolus* from FAO area 37 (Mediterranean Sea) were inspected and a total of 50 nematodes were selected to perform experiments of small-RNA isolation from L3 and from their released extracellular vesicles (= EVs), collected after incubations procedures described later. The vitality of L3s was evaluated based on their spontaneous movements, after the encapsulation removal. All the L3 selected for the study were mixed from different hosts, in order to have homogeneous samples and avoid any host-related batch effects. Moreover, L3 were identified at species level as belonging to *A. pegreffii*, using the molecular diagnostic key based on ITS PCR–RFLP procedure^51^. All L3 were washed repeatedly with filtered PBS before molecular procedures.

### Size and concentration analysis of EVs

The size distribution and particle concentration of the fraction recovered after the EVs enrichment procedure were measured using Nanoparticle Tracking Analysis. NTA was carried out using a Nanosight NS300 (Malvern Panalytical). Five measurements were performed with 60 s duration of each measurement and the data was analysed using NTA software version 3.4.

### RNA extraction, library preparation and RNA-sequencing

Three biological samples of L3 (5 pooled larve) and EVs (5 pooled larve) were used. Total small-RNA fraction from L3 were isolated using miRNeasy tissue kit (Qiagen, Hilden, Germany), according to manufacture instructions. EVs were obtained after incubating pool of L3 in 24 multiwell plates with filtered 1 ml of RPMI with 1% pen-strep for 24 h at 37 °C and 5%CO_2_. Surnatants were collected and Exoquick (System Bio) was used according to manufacturer’s instructions to obtain the exosome-enriched fractions (resuspended in 100ul of 20um filtered PBS). From these fractions, small-RNAs were obtained using miRNeasy Serum/Plasma kit (Qiagen, Hilden, Germany). Material was stored at -80 °C until used for RNA-seq. Concentration and purity of small-RNA were evaluated by determining the absorbance at 260 and 280 nm by a BioTek SynergyHT (Take3 Module) and using the Qubit4 (Thermo Fisher Scientific, USA). Then, RNA quality control and libraries preparation were performed at the EMBL Genomic Core Facility (EMBL, Heidelberg, DE), where a further RNA check was performed with an Agilent 2100 Bioanalyzer (Agilent Technologies). Small RNA libraries were prepared using the TruSeq Small RNA Sample preparation kit (Illumina). Fifty base pair, single end sequencing was performed on an Illumina HiSeq2500 platform.

### Reads mapping

Raw reads were first checked by FastQC^52^ and then trimmed using cutadapt 1.9.1^53^ to remove 3′ adapters and discard the reads shorter than 14 nucleotides. The processed reads from each biological sample were mapped to the *Anisakis simplex* genome assembly (= AS14) Bioproject PRJEB496 version downloaded from WormBase Parasite WBPS14^54^, using Bowtie^55^. Given the absence of an available genome assembly for *A. pegreffii*, *A. simplex* was selected as sibling species of *A. pegreffii*, showing different ecological traits and limited amount of genetic variation with respect to *A. pegreffii*. Reads were analysed for their frequency and size distribution.

Reads aligned to AS14 were also mapped to a collection of *Anisakis* spp. rRNA and ncRNAs (excluding hairpins and predicted miRNAs) retrieved from the available repositories GenBank, RNA Central and Rfam platform^56^. Reads unaligned to ribosomal RNAs were analysed for their size distribution as reported in Fig. 2 and then mapped (-n 0 -l 18 -a –best –strata -e 80 –norc) to two different lists. The first was composed by 153 putative miRNA precursors (see below) plus other *Anisakis* ncRNAs. Reads aligning to this list were used for the correlation analysis. The second list included 206 putative mature *Anisakis* miRNAs (compiled as described below); reads mapping to these miRNAs were used for the differential expression analysis and the heatmap construction.

### Prediction of *Anisakis pegreffii* miRNAs

In the absense of any previous information on miRNAs from *Anisakis* species, two independent and complimentary approaches were used to compile a list of putative *A. pegreffii* miRNAs to be used for mapping. In a first approach, precursors and mature miRNAs from *Ascaris suum* were retrieved from miRBase (release 22) and used to search potential orthologues in the genome of *A. simplex* by using the BLAST tool implemented in WormBase Parasite. *Ascaris suum* was chosen because represented the evolutionary closer parasitic nematode for which miRNA sequence information was available in public repositories, and *A. simplex* is the only species from the genus *Anisakis* with a sequenced genome. Hairpins were included according to inclusive parameters, when mature of the two species showed ≥ 85% identity on ≥ 80% length and max 2 mm in the seed (total mm max 6). This way a first list (“dataset1”) of *Anisakis* putative mature and precursor miRNAs was obtained. In a second approach, reads from our samples mapping to the *A. simplex* genome, and subtracted of those representing rRNAs and other ncRNAs (i.e., tRNAs, snoRNAs, snRNAs), were used to search the *A. simplex* genome by the miRNA prediction software miRDeep*^57^. This way a second list (“dataset2”) was obtained. The two datasets were compared in order to create an inclusive non-redundant inventory of putative hairpins (153) and mature (206) *Anisakis* miRNAs. Such lists were used for the different following analyses as described. Secondary structure predictions and minimal free energy calculations were performed using RNAfold^58^.

### Quantification and differential expression of miRNAs

Reads from *A. pegreffii* small-RNA libraries were mapped to the final list including 206 putative mature miRNAs and then used for the differential expression analysis. Reads with multiple highest score mappings were discarded. Expression values were calculated as count per millions (CPM, that is the number of reads mapping on a feature divided by the total number of mapped reads and multiplied by one million) and used for sample clustering. Reads mapping to miRNAs and hairpins included in the two datasets were assigned to mature miRNAs. Differential expression analysis of mature miRNAs with 1 CPM in at least two samples of each triplicate was performed using glmFIT and glmLRT functions provided by the edgeR software package^59,60^. Log2 Fold change (FC) and false discovery rates (FDR) were calculated to provide statistical validation (Supplementary Table 2) and inclusive parameters with moderate thresholds were considered for further analyses on DEGs miRNAs (FC > 1 and FDR < 0.05).

### miRNA validation by Real-Time Stem and Loop PCR amplification

Validation of a subset (10 miRNAs) of the putative miRNAs characterized in this study was performed by the Stem and Loop Reverse-Transcription Polymerase Chain Reaction technique^61^ using as template small-RNA L3 and enriched exosomal fractions of *A. pegreffii*. First-strand cDNA was generated using the SuperScript II Reverse Transcriptase (Invitrogen) according to manufacturer’s instruction and specific stem-loop primers (0.1 μM). Real time PCR amplifications were performed in a final volume of 20 μl including SYBR Green Master Mix (Applied Biosystem), specific forward and universal reverse primers (0.1 μM each) and 2 μl of cDNA. Amplification was as follows: initial holding stage of 2 min at 50 °C and 2 min at 95 °C followed by 40 cycles (30 s. 95 °C, 1 min 60 °C). All RT-qPCR reactions were performed in biological and technical triplicates. The subset of 10 miRNAs was selected according to their total abundance and their enrichment in EVs (ape-novel-miR-19, ape-miR-7-5p, ape-novel-miR-65, ape-novel-miR-27, ape-novel-miR-131, ape-miR-72-5p, ape-novel-miR-184, ape-miR-100a-5p, ape-lin-4-5p, ape-miR-1-3p). The sequence of all primers employed is provided in Supplementary Tables 4a and 4b.

### Target analysis and seed conservation

Prediction of putative *A. pegreffii* miRNAs gene targets was inferred using miRDB implementing MiRTarget bioinformatic tool^62^, an online database for miRNA target prediction and functional annotations, using human genome as query and indicating the related orthologue human miRNA. The top20 most abundant miRNAs and on those enriched in the exosomal fraction were considered for this analysis and only the targets with highest score and potential role in immune or inflammatory response were retained.

With the aim to explore conservation of seed region across parasitic helminthic lineages, we explored the amount of variation in *A. pegreffii* miRNAs in comparison to those available from other parasitic helminths, according to the supplementary data retrieved^21^, retaining only those with complete seed identity or maximum 1 mismatch. Complete list is available in Supplementary Table 3.

## Supplementary Information

Supplementary Information 1.

Supplementary Information 2.

Supplementary Information 3.

Supplementary Information 4.

Supplementary Information 5.

Supplementary Information 6.

Supplementary Information 7.

Supplementary Information 8.

## Data Availability

The small RNA-Seq datasets generated and analysed in the current study have been deposited in NCBI’s Gene Expression Omnibus and are accessible as Bioproject PRJNA786753. Other data generated during this study have been included as Supplementary Information.
