# Supplementary material for: A miRNAs catalogue from third-stage larvae and extracellular vesicles of Anisakis pegreffii provides new clues for host-parasite interplay
Source: Sci Rep. 2022 Jun 11;12:9667. doi: 10.1038/s41598-022-13594-3 (PMC9188560; doi:10.1038/s41598-022-13594-3)
Supplement: Supplementary file 1 — Supplementary Information 1. [file 41598_2022_13594_MOESM1_ESM.docx]

**Supplementary material**

**Supplementary Table T1:** list of *Anisakis pegreffii* miRNAs with indication of ID according to nomenclature, miRNA and hairpin sequence, ID of match with *Anisakis simplex* genome AS14, nucleotide position, correspondence to strand +/-, putative orthologue in miRBase, ID of putative orthologue mature (helminths other than *Ascaris suum* are indicated in red, *Homo sapiens* in bold) and miRNAs family according to RFAM platform.

**Supplementary Table T2**: Results from differential expression analysis with indication of miRNAs ID, logFC, logCPM and statistical support.

**Supplementary Table T3**: List of *Anisakis pegreffii* significantly abundant miRNAs in larvae and in exosomes, together with putative orthologues miRNAs from other parasitic helminths with conserved seed region, and human putative orthologues. The last two columns include *Anisakis pegreffii* miRNAs predictive targets in human genome according to miRDB and their ID according to NCBI database. Asterisks indicate a miRNAs enriched in exosomes, according to literature. (Underlined miRNAs are abundant both in L3 and in EVs list).

**Supplementary Table T4a:** miRNAs selected from L3 and EVs most abundant observations to validate with Stem and Loop RT-PCR technique. Specific codes of miRNAs, with the 6 terminal nucleotides are indicated in red (universal primer Stem and Loop = 5′GTCGTATCCAGTGCAGGGTCCGAGGTATTCGCACTGGATACGAC).

**Supplementary Table T4b:** specific forward primers for each miRNAs.

**Supplementary Figure 1:** NTA capture and analysis settings, with results for separated and averaged reads.

**Supplementary Figure 2:** Correlation and clustering. Multidimensional scaling plots based on Fold Change (top of the figure), matrix of correlation coefficient values (lower left part of the figure) and cluster dendrogram based on distance among samples (lower right part of the figure). Plots were generated using the plotMDS function implemented in the edgeR software package. Only small non-coding RNAs with CPM ≥ 1 in at least three replicates were used.

**Supplementary Figure 3:** Barplot of mean Ct values and standard errors obtained in stem and loop RT-PCR assays for selected miRNAs validated in larvae and extracellular vesicles samples.
