# Supplementary material for: A miRNAs catalogue from third-stage larvae and extracellular vesicles of Anisakis pegreffii provides new clues for host-parasite interplay
Source: Sci Rep. 2022 Jun 11;12:9667. doi: 10.1038/s41598-022-13594-3 (PMC9188560; doi:10.1038/s41598-022-13594-3)
Supplement: Supplementary file 4 — Supplementary Information 4. [file 41598_2022_13594_MOESM4_ESM.docx]

**Supplementary Table** **3**

| ***Anisakis pegreffii* miRNAs** | **miRNAs in other helminths** | **Human miRNAs** | **Target - putative role** | **GeneID NCBI** |
| --- | --- | --- | --- | --- |
| ***Top20 most abundant*** | | | | |
| **miR-100a-1-5p** | asu-miR-100b-5p  bma-miR-100a  bma-miR-100b  bma-miR-100c  bma-miR-100d  hpo-miR-100-5p* | hsa-miR-100-5p | **TRIB2** - interact and modulate the activity of signal transduction pathways in physiological and pathological processes. This Tribbles member induces apoptosis of cells mainly of the hematopoietic origin. | 28951 |
| **miR-1-3p** | asu-miR-1-3p  str-miR-1-3p  hco-miR-1-3p  hpo-miR-1-3p  fhe-miR-1-3p | hsa-miR-1-3p | **MMD** - monocyte to macrophage differentiation associated protein. | 23531 |
| **miR-71-5p** | asu-miR-71-5p  bma-miR-71  str-miR-71-5p  hpo-miR-71-5p  hco-miR-71 | No match | **NAA25** - auxiliary subunit of the heteromeric N-terminal acetyltransferase B complex | 80018 |
| **miR-9-5p** | asu-miR-9-5p  bma-miR-9-5p  str-miR-9-5p  hco-miR-9  hpo-miR-9-5p | hsa-miR-9-5p | **PRTG** - protogenin, member of the immunoglobulin superfamily | 283659 |
| **miR-100b-5p** | asu-miR-100b-5p  bma-miR-100b  bma-miR-100a  bma-miR-100d  bma-miR-100c  hpo-miR-100-5p | hsa-miR-100-5p  hsa-miR-10b-5p | **TRIB2** - interact and modulate the activity of signal transduction pathways in physiological and pathological processes. This Tribbles member induces apoptosis of hematopoietic cells. | 28951 |
| **lin-4-5p** | asu-lin-4-5p  bma-lin-4  str-lin-4-5p  hco-lin-4  hpo-lin-4-5p  cel-lin-4-5p | hsa-miR-125b | **ARID3B** - family of DNA-binding proteins with roles in embryonic patterning and cell lineage, cell cycle control, transcriptional regulation and chromatin structure modification.  **STARD13** - regulation of cytoskeletal reorganization, cell proliferation, cell motility, and acts as a tumor suppressor in hepatoma cells.  **BMF** - apoptotic activator  **FREM1** - co-receptor of the interleukin 1 receptor family contributing to the control of inflammatory response activation | 10620  90627  90427  158326 |
| **miR-81a** | asu-miR-81a  bma-miR-81  str-miR-81a-3p  hpo-miR-81-3p | No match | **SRSF11** - potential role in pre-mRNA processing | 9295 |
| **miR-5361-5p** | asu-miR-5361-5p  bma-miR-5361 | No match | **PRIKLE1**- potential negative regulator of the Wnt/beta-catenin signaling pathway. | 144165 |
| **miR-57-5p** | asu-miR-57-5p  bma-miR-57 | hsa-miR-10b-5p | **CADM2**- member of immunoglobulin superfamily. | 253559 |
| **miR-5358a-3p** | asu-miR-5358a-3p | No match | **TRIB2** - interact and modulate the activity of signal transduction pathways in physiological and pathological processes. This Tribbles member induces apoptosis of hematopoietic cells. | 28951 |
| **miR-50-5p** | asu-miR-50-5p  bma-miR-50  str-miR-50-5p | hsa-miR-137-5p | **NEUROD1**- neuronal differentiation factor 1. | 4760 |
| **miR-5364-3p** | asu-miR-5364-3p  bma-miR-5364 | No match | **STARD13** - regulation of cytoskeletal reorganization, cell proliferation, cell motility, and acts as a tumor suppressor in hepatoma cells. | 90627 |
| **miR-34-5p** | asu-miR-34-5p  bma-miR-34  str-miR-34a-5p  str-miR-34b-5p  str-miR-34c-5p  hpo-miR-34-5p | hsa-miR-34c-5p | **MD4 -** binds the p53 tumor suppressor protein and inhibits its activity by binding its transcriptional activation domain, overexpressed in a variety of human cancers. | 4194 |
| **miR-5360-5p** | asu-miR-5360-5p  bma-miR-5360 | No match | **CCR7**- expressed in various lymphoid tissues and activates B and T lymphocytes | 1236 |
| **novel-miR-124** | str-miR-240-3p | No match | **MAPK10** - involved in a variety of cellular processes, such as proliferation, differentiation, transcription regulation and development | 5602 |
| **miR-228-5p** | asu-miR-228-5p  bma-miR-228  hco-miR-228  hpo-miR-228-5p | No match | **PFN2** - regulation of actin polymerization in response to extracellular signals | 5217 |
| **miR-9-3p** | asu-miR-9-3p  bma-miR-9-3p  hpo-miR-9-3p  str-miR-9-3p | hsa-miR-9-3p | **ITGB1 -** cell adhesion and recognition in embryogenesis, hemostasis, tissue repair, immune response and metastatic diffusion of tumor cells. | 3688 |
| **miR-1822-3p** | asu-miR-1822-3p  bma-miR-1822  hpo-miR-1822-3p | No match | **GRM5 -** metabatropic glutamate receptor may be involved in the regulation of neural network activity and synaptic plasticity. | 2915 |
| **novel-miR-185** | No match | No match | **HIF1A -** master regulator of cellular and systemic homeostatic response to hypoxia by activating transcription of genes involved in energy metabolism, angiogenesis, and apoptosis. HIF-1 is essential in embryonic vascularization, tumor angiogenesis and pathophysiology of ischemic disease. | 3091 |
| **novel-miR-72** | No match | No match | **RAB5C -** members of the Rab protein (Ras superfamily) potential role in docking and/or fusion of vesicles | 5878 |
| ***Enriched in exosomes*** | | | | |
| **novel-miR-19** | No match | hsa-mir-1322  hsa-mir-4502 | **FAM83C -** involved in regulating MAPK signaling in cancer cells. | 128876 |
| **novel-miR-131** | No match | No match | **RORB -** DNA-binding protein that can bind to hormone response elements upstream of several genes (enhancer). | 6096 |
| **novel-miR-97** | No match | No match | **LINGO1 -** leucine rich repeat and Ig domain containing 1 | 84894 |
| **miR-7-5p** | asu-miR-7-5p  bma-miR-7  hco-miR-7  hpo-miR-7-5p | hsa-miR-7-5p  hsa-miR-3529-3p | **SPATA2 -** spermatogenesis associated 2 | 9825 |
| **novel-miR-65** | No match | No match | **GIPC3 -** gene required for postnatal maturation of the hair bundle and long-term survival of hair cells and spiral ganglion in the ear in mice. | 126326 |
| **novel-miR-57** | No match | hsa-mir-584-5p | **SLC20A2 -** role in phosphate homeostasis by mediating cellular phosphate uptake. The encoded protein also confers susceptibility to viral infection as a gamma-retroviral receptor. | 6575 |
| **miR-5353-3p** | asu-miR-5353-3p | No match | **NECAP2 -** member of the adaptin-ear-binding coat-associated protein family. Studies of a similar protein in rat suggest a role in clathrin-mediated endocytosis. | 55707 |
| **novel-miR-69** | No match | No match | **ZHX3 -** member of the zinc fingers and homeoboxes (ZHX) gene family. May function as a transcriptional repressor. | 23051 |
| **novel-miR-27** | asu-miR-750  sja-miR-750 | No match | **GNG2 -** gamma subunits of a guanine nucleotide-binding protein, involved in signaling mechanisms across membranes. | 54331 |
| **miR-72-5p** | asu-miR-72-5p  bma-miR-72  str-miR-72-5p  hpo-miR-72-5p  hco-miR-72 | hsa-miR-31-5p | **RSBN1 -** round spermatid basic protein 1 | 54665 |
| **novel-miR-184** | No match | hsa-mir-4448 | **STRADB:** master kinase that regulates cell polarity and energy-generating metabolism. It is essential for G1 cell cycle arrest mediated by this kinase. The protein encoded can interact with the X chromosome-linked inhibitor of apoptosis protein, enhancing the anti-apoptotic activity via the JNK1 pathway. | 55437 |
