# Supplementary material for: A miRNAs catalogue from third-stage larvae and extracellular vesicles of Anisakis pegreffii provides new clues for host-parasite interplay
Source: Sci Rep. 2022 Jun 11;12:9667. doi: 10.1038/s41598-022-13594-3 (PMC9188560; doi:10.1038/s41598-022-13594-3)
Supplement: Supplementary file 5 — Supplementary Information 5. [file 41598_2022_13594_MOESM5_ESM.docx]

**Supplementary Table T4a**

| **miRNA** | **6nt to merge with the universal primer S and L** |
| --- | --- |
| *ape-mir-1-3p* | 5′GTCGTATCCAGTGCAGGGTCCGAGGTATTCGCACTGGATACGAC**TACATA’3** |
| *ape-mir-7-5p* | 5′GTCGTATCCAGTGCAGGGTCCGAGGTATTCGCACTGGATACGAC**AACAAC** |
| *asi-novel-mir-19* | 5′GTCGTATCCAGTGCAGGGTCCGAGGTATTCGCACTGGATACGAC**TATCAT** |
| *asi-novel-mir-131* | 5′GTCGTATCCAGTGCAGGGTCCGAGGTATTCGCACTGGATACGAC**TCCTGA** |
| *asi-mir-100a-5p* | 5′GTCGTATCCAGTGCAGGGTCCGAGGTATTCGCACTGGATACGAC**AACACA** |
| *asi-mir-72-5p* | 5′GTCGTATCCAGTGCAGGGTCCGAGGTATTCGCACTGGATACGAC**TCAGCT** |
| *ape-lin4-5p* | 5′GTCGTATCCAGTGCAGGGTCCGAGGTATTCGCACTGGATACGAC**CTGTGA** |
| *ape-novel-mir-65* | 5′GTCGTATCCAGTGCAGGGTCCGAGGTATTCGCACTGGATACGAC**AATTCA** |
| *ape-novel-mir-27* | 5′GTCGTATCCAGTGCAGGGTCCGAGGTATTCGCACTGGATACGAC**CTTCCA** |
| *ape-novel-mir-184* | 5′GTCGTATCCAGTGCAGGGTCCGAGGTATTCGCACTGGATACGAC**GTATGA** |

**Supplementary Table T4b**

| **miRNA** | **primer forward** |
| --- | --- |
| *ape-miR-1-3p* | GCGTAAGTGGAATGTAAAGAAG |
| *ape-miR-7-5p* | CCTTGGAAGACTGGTGATTTT |
| *asi-novel-miR-19* | CACAGGGTGATGATGATGATG |
| *asi-novel-miR-131* | GGTTAGTGTAGCGGTTAGCAC |
| *asi-miR-100a-1-5p* | CCTTAACCCGTAGATCCGAACT |
| *asi-miR-72-5p* | GGCAGGCAAGATGTTGGCAT |
| *ape-lin4-5p* | CACACCCTCCCTGAGACCTCTG |
| *ape-novel-miR-65* | GGTCACCCATCTAGAGGAGCTG |
| *ape-novel-miR-27* | GGGGGGTTTCCAGATCTTCATA |
| *ape-novel-miR-184* | ATAACCGGCTCAGTGGTCTAGTG |
