# Supplementary figures and images for: A miRNAs catalogue from third-stage larvae and extracellular vesicles of Anisakis pegreffii provides new clues for host-parasite interplay
Source: Sci Rep. 2022 Jun 11;12:9667. doi: 10.1038/s41598-022-13594-3 (PMC9188560; doi:10.1038/s41598-022-13594-3)

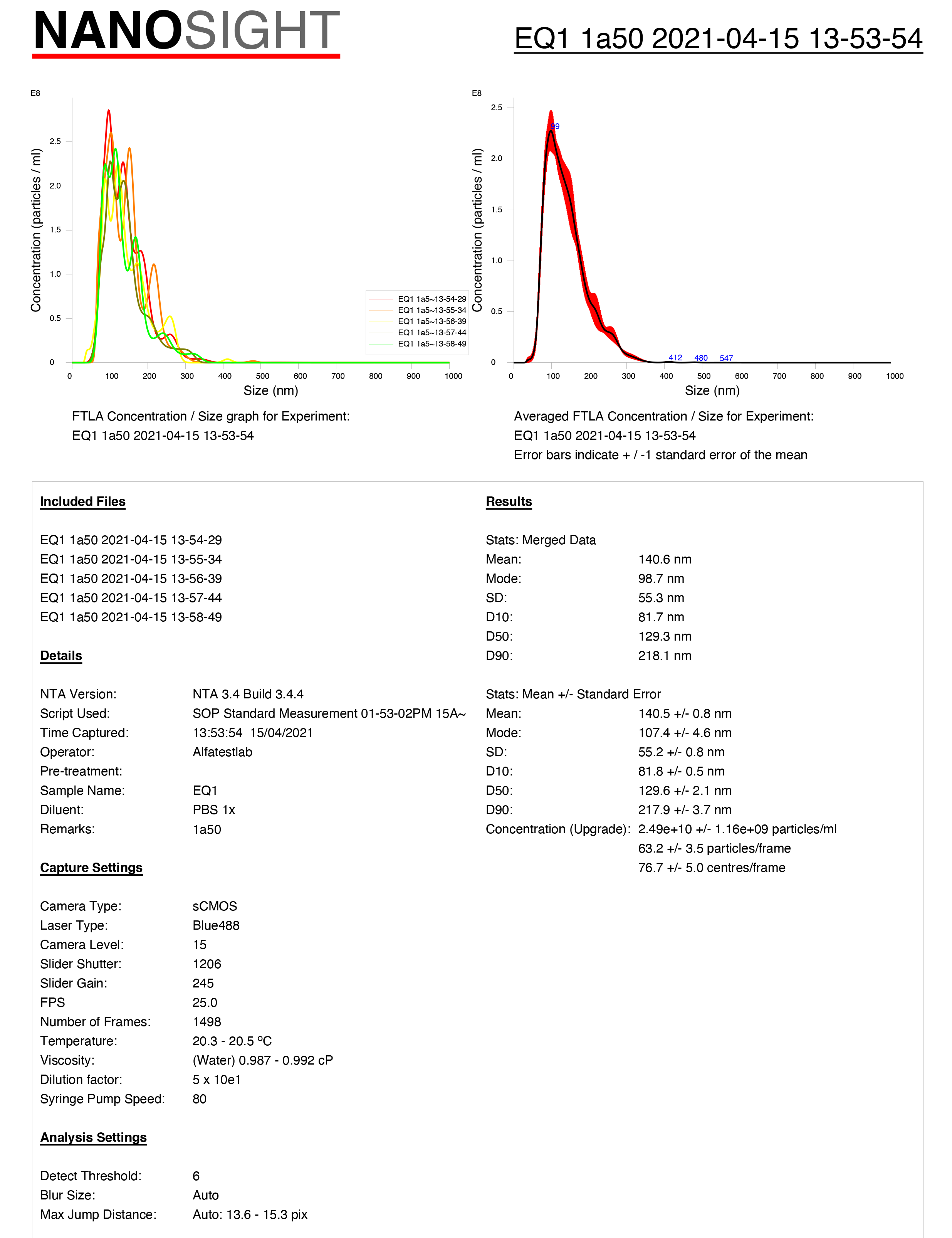

Supplement: Supplementary file 6 — Supplementary Information 6. [file 41598_2022_13594_MOESM6_ESM.tif]

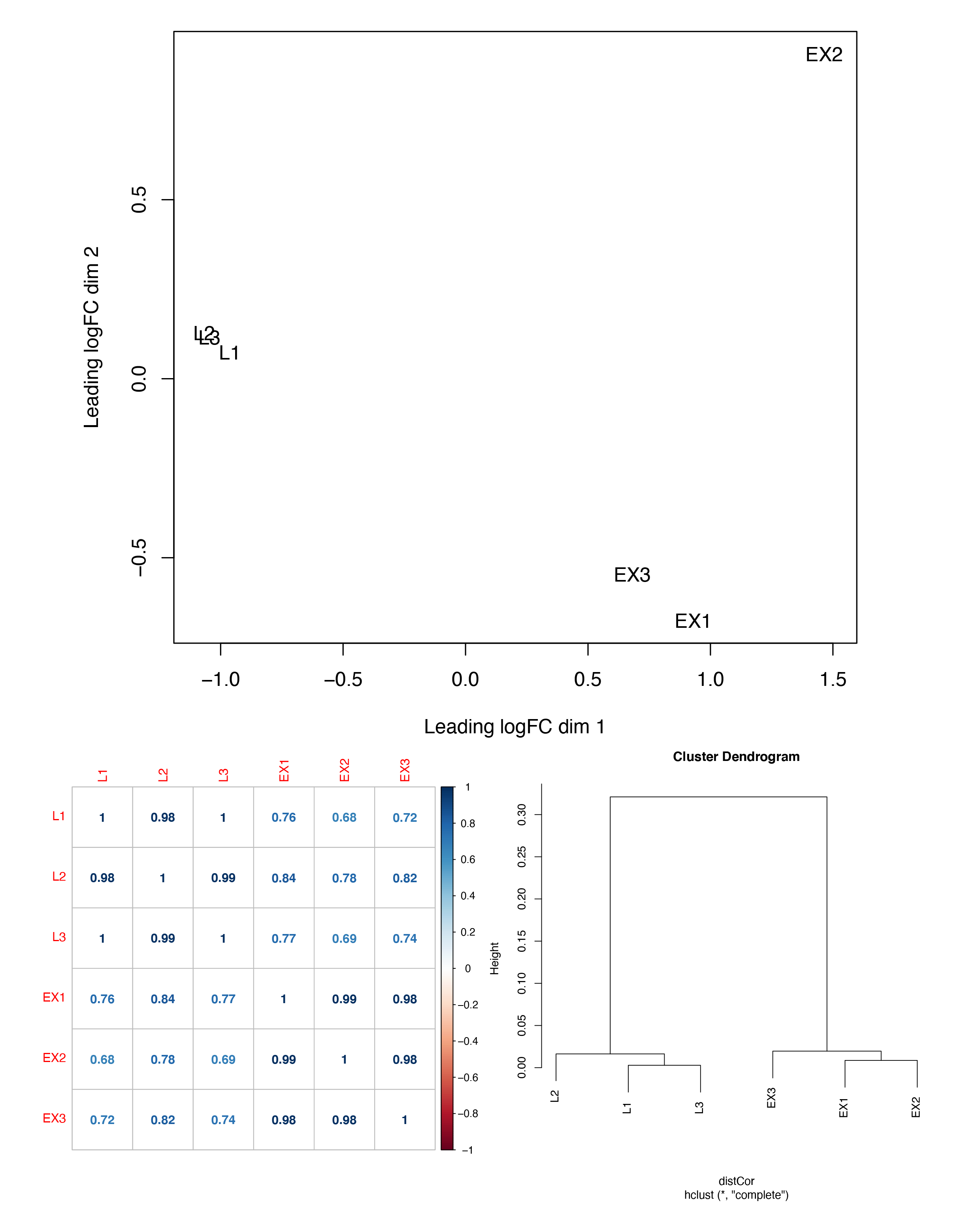

Supplement: Supplementary file 7 — Supplementary Information 7. [file 41598_2022_13594_MOESM7_ESM.tif]

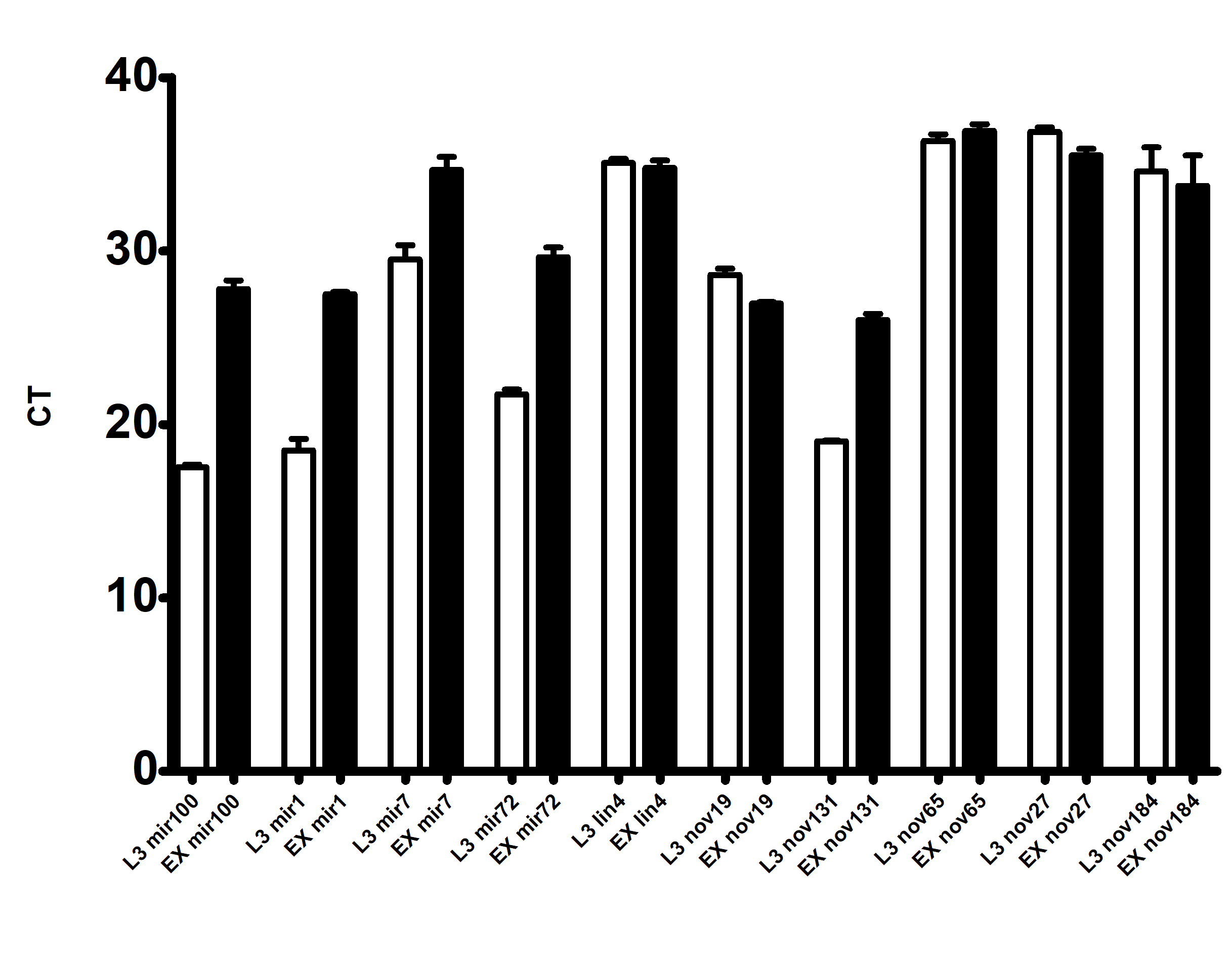

Supplement: Supplementary file 8 — Supplementary Information 8. [file 41598_2022_13594_MOESM8_ESM.jpg]
